# Comparison of RAPD, ISSR, and AFLP Molecular Markers to Reveal and Classify Orchardgrass (*Dactylis glomerata* L.) Germplasm Variations

**DOI:** 10.1371/journal.pone.0152972

**Published:** 2016-04-12

**Authors:** Rita Costa, Graça Pereira, Inmaculada Garrido, Manuel María Tavares-de-Sousa, Francisco Espinosa

**Affiliations:** 1 Unidade Estratégica de Investigação e Serviços de Biotecnologia e Recursos genéticos, INIAV-Pólo de Elvas, Estrada Gil Vaz, 6. 7351–901, Elvas, Portugal; 2 Área de Fisiología Vegetal, Facultad de Ciencias, Universidad de Extremadura, Avda. de Elvas s/n. E-06006, Badajoz, Spain; Chinese Academy of Medical Sciences, Peking Union Medical College, CHINA

## Abstract

Three different DNA-based techniques, Random Amplified Polymorphic DNA (RAPD), Inter Simple Sequence Repeat (ISSR) and Amplified Fragment Length Polymorphism (AFLP) markers, were used for fingerprinting *Dactylis glomerata* genotypes and for detecting genetic variation between the three different subspecies. In this study, RAPD assays produced 97 bands, of which 40 were polymorphic (41.2%). The ISSR primers amplified 91 bands, and 54 showed polymorphism (59.3%). Finally, the AFLP showed 100 bands, of which 92 were polymorphic (92%). The fragments were scored as present (1) or absent (0), and those readings were entered in a computer file as a binary matrix (one for each marker). Three cluster analyses were performed to express–in the form of dendrograms–the relationships among the genotypes and the genetic variability detected. All DNA-based techniques used were able to amplify all of the genotypes. There were highly significant correlation coefficients between cophenetic matrices based on the genetic distance for the RAPD, ISSR, AFLP, and combined RAPD-ISSR-AFLP data (0.68, 0.78, 0.70, and 0.70, respectively). Two hypotheses were formulated to explain these results; both of them are in agreement with the results obtained using these three types of molecular markers. We conclude that when we study genotypes close related, the analysis of variability could require more than one DNA-based technique; in fact, the genetic variation present in different sources could interfere or combine with the more or less polymorphic ability, as our results showed for RAPD, ISSR and AFLP markers. Our results indicate that AFLP seemed to be the best-suited molecular assay for fingerprinting and assessing genetic relationship among genotypes of *Dactylis glomerata*.

## Introduction

*Dactylis glomerata* L. is a highly variable perennial forage grass. It is extensively cultivated in all of the world's temperate and subtropical growing regions [1]. This, of course, includes Portugal in the Iberian Peninsula [2] where it grows in the sandy soils of the coast, the shallow soils of the interior, and is cultivated or natural in the grasslands of the north [3]. There are 4 diploid (2n = 2x = 14), 16 tetraploid (2n = 4x = 28), and 1 hexaploid (2n = 6x = 42) subspecies of *Dactylis glomerata* [4]. The different degrees of ploidy reflect different adaptations to soil and climate. Alone or together with legumes, natural or cultivated as an irrigated or dryland crop, it is one of the most important grasses for grazing and hay [5]. Moreover, the rapid growth of its root system makes it especially important for use a cover to protect against surface erosion and to restore degraded soils [6]. The diploid subspecies have a more restricted geographical and ecological distribution than the tetraploid. The present study considers the tetraploid subspecies *glomerata* and *hispanica*, and the diploid subspecies *lusitanica* whose distribution has been confined to small areas by deforestation and agriculture [2]. In order for the indigenous genetic resources of the wild germplasm to be exploited and conserved for breeding purposes [7], it is imperative to assess the genetic variability of wild accessions.

Variations in orchardgrass's morphological features, distribution patterns, and adaptive and agronomic characters are well documented [5[, [8], [9], [10]. Geographically distinct populations can differ in their levels of genetic diversity or in the spatial distribution of that diversity [2], [7]DNA profiling techniques that have been successfully used to assess the genetic diversity and relatedness of orchardgrass germplasm include RAPD (Random Amplified Polymorphic DNA) [11], [12], [13], [14], AFLP (Amplified Fragment Length Polymorphism) [1], [15], ISSR (Inter-Simple Sequence Repeat) [7], [13], [14], and SSRs (Simple Sequences Repeat) [16] markers. Even though these studies demonstrated the usefulness of DNA profiling in assessing genetic differences in orchardgrass, only one was focused on the genetic diversity and relationships in Portuguese populations [13]. Molecular markers provide a direct measure of genetic diversity, and complement measures based on agronomic traits or geographic origins. Technically however, the different molecular markers are not equal in terms of cost, speed, amount of DNA needed, labour, and degree of polymorphism. RAPD analysis is simple, rapid, and has the ability to detect extensive polymorphisms. It is particularly well-suited to DNA fingerprinting [17] although it does suffer from a certain lack of reproducibility due to mismatch annealing [18]. AFLP analysis is robust, and reveals high numbers of reproducible polymorphic bands with just a few primer combinations [19]. Both of these techniques are fast, inexpensive, and do not require prior sequence information [20]. ISSR markers comprise a few highly informative multi-allelic loci. They provide highly discriminating information with good reproducibility, and are relatively abundant [21], [22]. Of these three techniques, while AFLP is the most labour intensive and time-consuming, it is also the most reliable

Germplasm improvement and genetic diversity is the key to durable and sustained production of *Dactylis glomerata*. There have been comparisons of molecular markers for estimating genetic diversity and also the combined analysis from all marker systems in different species [23], [24], [25], [26], [27]. Madesis et al. [16] in *Dactylis glomerata* have studied the genetic diversity and structure natural populations using microsatellite-based markers. The objectives of the present study were therefore: (i) the molecular characterization of the germplasm of wild Portuguese orchardgrass using molecular markers, and (ii) to compare the level of information provided by RAPD, ISSR, and AFLP markers for the assessment of genetic similarities.

## Material and Methods

### Species, study locations, and sampling

Ninety-one accessions were obtained in a plant-collecting expedition in Portugal, and placed in a field at the Plant Breeding Station, Elvas, Portugal. All of the material was evaluated in that same place. In the expedition, ten regions were explored from the north to the south of Portugal. A diverse range of habitats was sampled, covering different altitudes, management systems, and ecological conditions including semi-natural and unmanaged wild grasslands (**Table 1**). At each localization, seeds were randomly collected from 20 plants of *Dactylis glomerata*. The plant recollection did not require specific permission, and did not involve endangered species.

**Table 1:** Genotypes, subspecies, and geographic distribution of *Dactylis glomerata*.

Genotype code	Subspecies	Origin	Latitude (°N)	Longitude (°W)
1	*glomerata*	Beja	37° 46’ 12”	8° 01’ 12”
2	*glomerata*	Beja	37° 46’ 12”	8° 01’ 12”
3	*hispanica*	Beja	37° 57’ 36”	7° 37’ 48”
4	*hispanica*	Beja	38° 08’ 20”	7° 27’ 00”
5	*hispanica*	Beja	37° 41’ 24”	8° 05’ 24”
6	*glomerata*	Beja	37° 41’ 24”	8° 05’ 24”
7	*glomerata*	Faro	37° 08’ 24”	8° 29’ 24”
8	*glomerata*	Beja	37° 38’ 24”	8° 39’ 36”
9	*glomerata*	Évora	38° 27’ 00”	7° 29’ 24”
10	*glomerata*	Évora	38° 27’ 00”	7° 29’ 24”
11	*hispanica*	Beja	37° 38’ 24”	8° 39’ 36”
12	*glomerata*	Évora	38° 25’ 12”	7° 31’ 48”
13	*hispanica*	Évora	38° 27’ 00”	8° 39’ 36”
14	*hispanica*	Beja	37° 38’ 24”	8° 39’ 36”
15	*glomerata*	Beja	37° 41’ 24”	8° 05’ 24”
16	*glomerata*	Beja	37° 56’ 24”	7° 36’ 00”
17	*hispanica*	Évora	38° 41’ 59”	7° 23’ 59”
18	*hispanica*	Évora	38° 41’ 59”	7° 23’ 59”
19	*hispanica*	Faro	37° 08’ 24”	8° 29’ 24”
20	*glomerata*	Beja	37° 56’ 24”	7° 36’ 00”
21	*glomerata*	Évora	38° 17’ 60”	7° 15’ 36”
22	*glomerata*	Évora	38° 17’ 60”	7° 15’ 36”
23	*glomerata*	Évora	38° 26’ 24”	7° 22’ 48”
24	*hispanica*	Évora	38° 26’ 24”	7° 22’ 48”
25	*hispanica*	Beja	37° 57’ 36”	7° 37’ 48”
26	*glomerata*	Évora	38° 49’ 48”	7° 50’ 24”
27	*hispanica*	Évora	38° 17’ 60”	7° 15’ 36”
28	*hispanica*	Beja	37° 56’ 24”	7° 36’ 00”
29	*glomerata*	Beja	37° 41’ 24”	8° 05’ 24”
30	*glomerata*	Beja	37° 41’ 24”	8° 05’ 24”
31	*glomerata*	Beja	37° 41’ 24”	8° 05’ 24”
32	*glomerata*	Currie, commercial variety		
33	*glomerata*	Beja	37° 46’ 12”	8° 01’ 12”
34	*hispanica*	Beja	37° 48’ 36”	8° 17’ 24”
35	*hispanica*	Santarém	39° 22’ 48”	8° 01’ 48”
36	*glomerata*	Currie, commercial variety		
37	*glomerata*	Currie, commercial variety		
38	*glomerata*	Beja	37° 38’ 24”	8° 39’ 36”
39	*hispanica*	Évora	38° 17’ 60”	7° 15’ 36”
40	*glomerata*	Beja	37° 41’ 24”	8° 05’ 24”
41	*glomerata*	Beja	37° 41’ 24”	8° 05’ 24”
42	*glomerata*	Évora	38° 53’ 24”	8° 01’ 12”
43	*glomerata*	Faro	37° 13’ 48”	8° 17’ 24”
44	*hispanica*	Évora	38° 56’ 24”	8° 09’ 36”
45	*hispanica*	Beja	37° 42’ 36”	7° 36’ 36”
46	*hispanica*	Beja	37° 38’ 24”	8° 39’ 36”
47	*glomerata*	Beja	37° 38’ 24”	8° 39’ 36”
48	*hispanica*	Évora	38° 17’ 60”	7° 15’ 36”
49	*lusitanica*	Évora	38° 46’ 48”	7° 25’ 12”
50	*hispanica*	Beja	37° 38’ 24”	8° 39’ 36”
51	*hispanica*	Beja	37° 38’ 24”	8° 39’ 36”
52	*hispanica*	Beja	37° 48’ 00”	7° 51’ 00”
53	*hispanica*	Beja	37° 48’ 00”	7° 51’ 00”
54	*hispanica*	Coimbra	40° 09’ 00”	7° 51’ 00”
55	*hispanica*	Évora	38° 34’ 12”	7° 54’ 36”
56	*hispanica*	Évora	38° 34’ 12”	7° 54’ 36”
57	*hispanica*	Beja	38° 03’ 36”	8° 07’ 12”
58	*hispanica*	Beja	38° 03’ 36”	8° 07’ 12”
59	*hispanica*	Beja	37° 36’ 00”	8° 39' 00”
60	*hispanica*	Setúbal	38° 08’ 20”	8° 29’24”
61	*glomerata*	Setúbal	38° 08’ 20”	8° 29’24”
62	*glomerata*	Australia		
63	*glomerata*	Leiria	39° 40’ 48”	8° 53’ 24”
64	*glomerata*	Évora	38° 30’ 00”	8° 09’ 36”
65	*hispanica*	Beja	37° 25’ 48”	8° 27’ 00”
66	*glomerata*	Currie, commercial variety		
67	*glomerata*	Vila Real	41° 09’ 36”	7° 47’ 24”
68	*glomerata*	Vila Real	41° 09’ 36”	7° 47’ 24”
69	*glomerata*	Faro	37° 05’ 24”	8° 45’ 36”
70	*glomerata*	Faro	37° 05’ 24”	8° 45’ 36”
71	*glomerata*	Faro	37° 05’ 24”	8° 45’ 36”
72	*hispanica*	Vila Real	41° 09’ 36”	7° 47’ 24”
73	*glomerata*	Vila Real	41° 09’ 36”	7° 47’ 24”
74	*hispanica*	Bragança	41° 27’ 36”	7° 15’ 36”
75	*hispanica*	Faro	37° 14’ 24”	8° 02' 24”
76	*hispanica*	Faro	37° 15’ 00”	7° 34’ 12”
77	*hispanica*	Beja	37° 48’ 36”	8° 17’ 24”
78	*lusitanica*	Évora	38° 46’ 48”	7° 25’ 12”
79	*glomerata*	Faro	37° 11’ 24”	8° 25’ 48”
80	*hispanica*	Bragança	41° 35’ 24”	7° 13’ 48”
81	*hispanica*	Évora	38° 30’ 00”	8° 09’ 36”
82	*glomerata*	Aveiro	40° 33’ 00”	8° 40’ 48”
83	*glomerata*	Faro	37° 07’ 48”	7° 39’ 00”
84	*hispanica*	Faro	37° 13’ 48”	7° 46’ 48”
85	*hispanica*	Beja	37° 48’ 36”	8° 17’ 24”
86	*hispanica*	Faro	37° 07’ 48”	7° 39’ 00”
87	*glomerata*	Beja	37° 40’ 48”	8° 33’ 00”
88	*hispanica*	Setúbal	38° 08’ 20”	8° 29’ 24”
89	*glomerata*	Faro	37° 25’ 48”	8° 27’ 00”
90	*hispanica*	Faro	37° 14’ 24”	8° 02’ 24”
91	*glomerata*	Faro	37° 15’ 00”	7° 34’ 12”
92	*hispanica*	Faro	37° 11’ 24”	8° 25’ 48”
93	*glomerata*	Faro	37° 11’ 24”	8° 25’ 48”
94	*glomerata*	Faro	37° 14’ 24”	8° 02’ 24”
95	*glomerata*	Currie, commercial variety		
96	*hispanica*	Faro	37° 13’ 48”	7° 46’ 48”
97	*hispanica*	Bragança	41° 35’ 24”	7° 13’ 48”
98	*glomerata*	Bragança	41° 35’ 24”	7° 13’ 48”
99	*hispanica*	Bragança	41° 27’ 36”	7° 15’ 36”
100	*hispanica*	Faro	37° 14’ 24”	8° 02’ 24”

### Molecular diagnostics

The material was subjected to molecular evaluations to determine its DNA-based diversity. To minimize the variance, we use approximately the same number of markers (that is RAPD—97 markers; ISSR—91 markers; AFPL—100 markers). The details of each technique are given in the following subsections.

### DNA extraction

DNA was isolated from young leaves following the CTAB (cetyl-trimethyl-ammonium-bromide) protocol [28], pooling samples from 4 genotypes from each accession. To remove RNA, the protocol includes treatment with RNase A at 37°C for 1 hour. The quality and quantity of the purified DNA was checked by 1% agarose gel electrophoresis using uncut lambda (λ) DNA as standard. The DNA solution was diluted to 20 ng/μl for PCR analysis.

### RAPD analysis

For the RAPD analysis, the total reaction volume was 25 μl containing 20 ng template DNA, 0.16 mM of each dNTP, 0.4 μM decanucleotide primer, 1 U Taq DNA polymerase (Pharmacia Biotech), 1.5 mM MgCl_2_, and 1× PCR buffer (10 mM Tris-HCl pH 9.0, 50 mM KCl). The PCR amplification was carried out in a thermocycler (Biometra UNO II) programmed as follows: an initial denaturation step of 90 s at 94°C followed by 35 cycles consisting of a denaturation step of 30 sec at 94°C, an annealing step of 1 min at 36°C, and an extension step of 2 min at 72°C. The last cycle was followed by 10 min at 72°C to ensure the completion of the primer extension reaction. An aliquot of 15 μl of the amplified products was subjected to electrophoresis in a 2% agarose gel cast in 1× TBE and run in 0.5× TBE at 100 V for 2.5–3.0 h. A digital image of the ethidium bromide-stained gel was captured using a Kodak Science 120ds Imaging System, and the bands were scored from the image displayed on the monitor. GeneRuler 100bp DNA ladder (MBI, Fermentas) was used to determine the size of the ISSR fragments. The 26 Operon primers used are listed in **Table 2**. All the reactions were repeated at least twice to check the reproducibility of the banding patterns.

**Table 2:** Primer´s sequence used and extent of polymorphism.

Marker	Primer	Primer´s Sequence (5´- 3´)	Total no. of loci/bands	No. of polymorphic loci/bands	% of Polymorphism (P)	Marker	Primer	Primer´s Sequence (5´- 3´)	Total no. of loci/bands	No. of polymorphic loci/bands	% of Polymorphism (P)
RAPD	OPA 02	TGCCGAGCTG	4	1	25.0	ISSR	01	(CA)_8_RG	5	4	80.0
	OPA 03	AGTCAGCCAC	6	0	0.0		03	(GA)_8_YT	9	9	100.0
	OPA 04	AATCGGGCTG	5	5	100.0		04	(GA)_8_YC	7	6	85.7
	OPA 05	AGGGGTCTTG	5	1	20.0		05	(GA)_8_YG	3	1	33.3
	OPA 07	GAAACGGGTG	1	0	0.0		06	(AG)_8_YT	5	5	100.0
	OPA 08	GTGACGTAGG	4	3	75.0		07	(AG)_8_YC	6	3	50.0
	OPA 09	GGGTAACGCC	4	3	75.0		09	(AC)_8_YG	1	0	0.0
	OPA 11	CAATCGCCGT	9	6	66.7		11	(GT)_8_YG	2	1	50.0
	OPA 13	CAGCACCCAC	6	2	33.3		12	(AG)_8_YG	5	1	20.0
	OPA 15	TTCCGAACCC	1	0	0.0		14	(AG)_8_T	5	5	100.0
	OPA 17	GACCGCTTGT	2	0	0.0		15	(AG)_8_C	6	3	50.0
	OPA 18	AGGTGACCGT	5	4	80.0		17	(GA)_8_T	6	5	83.3
	OPA 19	CAAACGTCGG	5	0	0.0		19	(GA)_8_A	3	2	66.7
	OPA 20	GTTGCGATCC	1	1	25.0		21	(TC)_8_C	1	0	0.0
	OPL 02	TGGGCGTCAA	1	0	0.0		23	(CT)_8_RC	2	1	50.0
	OPL 03	CCAGCAGCTT	6	3	75.0		25	(TG)_8_RC	1	0	0.0
	OPL 05	ACGCAGGCAC	3	0	0.0		27	BDB(CA)_7_	9	3	33.3
	OPL 07	AGGCGGGAAC	3	0	0.0		28	DBD(AC)_7_	6	1	16.7
	OPL 08	AGCAGGTGGA	4	3	75.0		29	VHV(GT)_7_	3	1	33.3
	OPL 12	GGGCGGTACT	4	2	33.3		30	HVH(TG)_7_	1	0	0.0
	OPL 14	GTGACAGGCT	2	0	0.0		31	(AG)_8_VC	2	1	50.0
	OPL 16	AGGTTGCAGG	2	0	0.0		32	CCC(GT)_7_	3	2	66.7
	OPL 18	ACCACCCACC	4	2	33.3	AFLP	ACC/CTT		33	33	33.0
	OPN 12	CACAGACACC	2	0	0.0		ACC/CAT		29	25	25.0
	OPN 13	AGCGTCACTC	3	2	33.3		ACC/CAG		32	32	32.0
	OPN 15	CAGCGACTGT	5	2	33.3		ACT/CAA		6	2	2.0

### ISSR analysis

For the ISSR analysis, 22 ISSR primers (**Table 2**) described by Farinhó et al. [29] were screened using a few DNA samples. The total reaction volume was 20 μl containing 40 ng template DNA, 0.2 mM of each dNTP, 0.5 μM decanucleotide primer, 1 U Taq DNA polymerase (Pharmacia Biotech), 1.5 mM MgCl_2_, and 1× PCR buffer (10 mM Tris-HCl pH 9.0, 50 mM KCl). The PCR amplification was carried out in the Biometra UNO II thermocycler programmed as follows: an initial denaturation step of 4 min at 94°C followed by 40 cycles consisting of a denaturation step of 30 s at 94°C, a primer annealing step of 45 s at 52°C, and an extension step of 2 min at 72°C. The last cycle was followed by 7 min at 72°C for final extension. The amplification products were analysed by electrophoresis in 2% agarose gel in 0.5× TBE buffer and detected by ethidium bromide staining. The GeneRuler 100bp DNA ladder (MBI, Fermentas) was used to determine the size of the ISSR fragments.

### AFLP analysis

The AFLP analysis was performed used the "AFLPTM Analysis System I" kit (Life Technologies) following Vos et al. [30]. Briefly, approximately 300 ng of genomic DNA of each accession was double digested with 3 U *Eco*RI and 3 U *Mse*I restriction enzymes. Adapters for the two enzymes (sequence for *Eco*RI: 5'-CTCGTAGACTGCGTACC/CATCTGACGCATGGTTAA-5'; sequence for *Mse*I: 5'-GACGAYGAGTCCTGAG/TACTCAGGACTCAT-5') were then ligated to the ends of restriction fragments using T4 DNA ligase (Promega). The sequences of the *Eco*RI and *Mse*I primers are 5'-GACTGCGTACCAATTC NNN and 5'GATGAGTCCTGAGTAA NNN, respectively (N = selective nucleotide). The pre-amplification consisted of 30 s at 94°C followed by 30 cycles of 30 s at 94°C, 1 min at 56°C, and 1 min at 72°C. The product DNA was diluted 50-fold with sterile nanopure water, and then used as template for the selective amplification. Four *Eco*RI:*Mse*I AFLP selective primer combinations were chosen for the selective amplification: ACC/CTT, ACC/CAT, ACC/CAG, and ACT/CAA (**Table 2**). The *Eco*RI primer was radiolabeled with γ-[33P] ATP. All the primers had three selective nucleotides. The selective amplification PCR reaction was performed with a final volume of 10 μl containing 1× PCR buffer, 1.5 mM MgCl_2_, 2 mM dNTP, 15 ng each of *Eco*RI and *Mse*I primers, 0.25 U Taq polymerase, and 2.5 μl pre-amplified template DNA. The following touchdown thermal profile was used: one cycle of 2 min at 94°C; 12 touchdown cycles of 30 s at 94°C, 30 s at 65°C (-0.7°C per cycle), 60 s at 72°C; and 23 cycles of 30 s at 94°C, 30 s at 56°C, 60 s at 72°C. All PCR reactions were carried out in the BIOMETRA UNO II thermocycler. An aliquot of 5 μl of selectively amplified PCR products was mixed with 5 μl loading buffer (98% formamide, 10 mM EDTA, 0.25% xylene cyanol, and 0.25% bromophenol blue), and denatured for 5 min at 94°C. The AFLP fragments were separated by denaturing 6% polyacrylamide gel electrophoresis (PAGE) with 8.3 M urea, and 1× TBE buffer. As standards for amplified DNA bands were used two molecular size markers 30 and 300 bp AFLP DNA Ladder (Gibco, Life Technologies) and a GeneRuler DNA Ladder Mix (MBI Fermentas), previously radiolabeled with γ-[33P] ATP. Gels were run at 70 W for 2 h using A BIOMETRA Model S2001 electrophoresis system with an Amersham Pharmacia Biotech EPS 3501 power supply, and visualised by autoradiography. The photographic plates were developed in accordance with the manufacturer's instructions (Kodak GBX).

### Data analysis

Amplification products were scored as present (1) or absent (0), compiling the data as a binary matrix. Only clear bands were scored. The level of polymorphism for each primer was represented by the percentage of polymorphic variable loci relative to all the loci analysed. Genetic variability and population genetic diversity indices, and relative genetic similarity coefficients were calculated as described by Nei [31] and Nei and Li [32]. Calculations were performed using the program POPGENE v. 1.31 with Microsoft Excel [33].

Similarity indices were computed using the Jaccard coefficient [34] to estimate relationships between accessions. Dendrograms were constructed using the unweighted pair-group method with arithmetic means (UPGMA) for clustering. For each of the dendrograms obtained from the RAPD, ISSR, AFLP, and RAPD+ISSR+AFLP combination data, a cophenetic matrix was generated using NTSYS-pc [35]. The Mantel significance test [36] was used to compare each pair of the similarity matrices produced. In addition, for each matrix, the average similarity was calculated for all pairwise comparisons within each of the intraspecific groups, and for all between-group pairwise comparisons.

## Results and Discussion

### Fingerprinting

In order to increase the confidence level of the fragments included in the RAPD, ISSR, and AFLP matrices, we scored very conservatively, excluding weak bands or bands that were ambiguous for some genotypes. We were very aware of the possibility with this approach of losing more than one band carrying useful information, but the aim was to obtain reproducible and clear data.

All the techniques tested in this study were able to uniquely fingerprint each of the 91 orchardgrass accessions. The number of assay units for each marker system varied from only 4 primer combinations for AFLP to 26 RAPD primers (**Tables 2 and 3**). The number of bands scored ranged from 91 for ISSR to 100 for AFLP. With only 4 primer combinations, AFLP gave the greatest number of bands scored (100), 92% of which were polymorphic. In contrast, for the case of RAPD, 97 bands were scored, with only 41.2% polymorphic. The ISSR case was intermediate, with 59.3% of its 91 bands being polymorphic. With respect to the number of polymorphic bands per assay unit, the highest value was with AFLP (25.00), with the RAPD and ISSR values far lower (3.73 and 4.14, respectively).

**Table 3:** Comparison of the RAPD, ISSR and AFLP banding pattern.

Marker	Number of assay units	Total no. of loci/bands	No. of polymorphic loci/bands	% of Polymorphism (P)	No. of loci/bands per assay unit
RAPD	26 primers	97	40	41.2%	3.73
ISSR	22 primers	91	54	59.3%	4.14
AFLP	4 primers combinations	100	92	92.0%	25.00

### Genetic variability

**The estimated** genetic variability within the orchardgrass subspecies studied (*glomerata*, *lusitanica*, *hispanica*), and the genetic diversity indices for the three combined are shown in **Table 4**. The Jaccard similarity coefficients between the 91 accessions were high for RAPD (ranging from 0.82 to 0.99), fairly high for ISSR (0.74–1.00), and low for AFLP (0.35–0.68). The lowest value was between accessions 6 and 21 (AFLP) which were collected from the provinces of Beja and Évora in southwestern Portugal. The highest value (1.00) was between accessions 9 and 67 from the provinces of Évora and Vila Real in southwestern and northern Portugal, respectively.

**Table 4:** Genetic variability within subspecies of orchardgrass (glomerata, lusitanica, hispanica) detected by RAPD, ISSR, and AFLP (A), and population genetic diversity indices for the three combined (B).

A															
subspecies	RAPD					ISSR					AFLP				
	P	n_a_ ± SD	n_e_ ± SD	H ± SD	I ± SD	P	n_a_ ± SD	n_e_ ± SD	H ± SD	I ± SD	P	n_a_ ± SD	n_e_ ± SD	H ± SD	I ± SD
*glomerata*	41.24	1.41±0.50	1.33±0.42	0.18±0.22	0.26±0.32	29.34	1.59±0.49	1.45±0.40	0.25±0.21	0.36±0.31	92.00	1.92±0.27	1.31±0.31	0.20±0.15	0.33±0.21
*lusitanica*	17.53	1.18±0.38	1.12±0.27	0.07±0.16	0.11±0.23	18.68	1.19±0.39	1.13±0.28	0.08±0.16	0.11±0.24	16.00	1.16±0.37	1.11±0.26	0.07±0.15	0.01±0.22
*hispanica*	41.24	1.41±0.49	1.30±0.39	0.17±0.21	0.25±0.30	56.04	1.56±0.50	1.42±0.41	0.23±0.22	0.34±0.31	93.00	1.93±0.26	1.30±0.30	0.20±0.15	0.32±0.20
B															
	**RAPD**					ISSR					AFLP				
	H_T_	H_S_	D_ST_	G_ST_	N_m_	H_T_	H_S_	D_ST_	G_ST_	N_m_	H_T_	H_S_	D_ST_	G_ST_	N_m_
*D*.*glomerata*	0.17	0.14	0.03	0.15	2.83	0.23	0.19	0.04	0.20	2.00	0.20	0.15	0.05	0.22	1.77

**P**: percentage of polymorphic bands; **n**_**a**_: observed number of alleles; **n**_**e**_: effective number of alleles; **H**: Nei´s gene diversity; **I**: Shannon´s index; **H**_**T**_: total genetic diversity; **H**_**S**_: intra-population genetic diversity; **D**_**ST**_: inter-population genetic diversity; **G**_**ST**_: genetic differentiation; **N**_**m**_: estimation of gene flow.

To get a more detailed view of the distribution of genetic variation within different groups, the Shannon index (I) was calculated for the total gene diversity within the subspecies (*glomerata*, *lusitanica*, *hispanica*). The respective values were: 0.26, 0.11, 0.25 for RAPD; 0.36, 0.11, 0.34 for ISSR; and 0.33, 0.10, 0.32 for AFLP (**Table 4A**). The intra-population genetic diversity is much higher than inter-population (**Table 4B**). Similar results were described by Madesis et al. [16] in *Dactylis glomerata* and Manners et al. [26] in *Vanda coerulea* showing that was higher genetic diversity within population that inter-population. Nm values indicate that gene flow is occurring among the three subspecies.

There were highly significant correlation coefficients between the cophenetic matrices based on the genetic distance for the RAPD, ISSR, AFLP, and combined RAPD-ISSR-AFLP data (0.68, 0.78, 0.70, and 0.70, respectively). Similar results have been described in various species by Biswas et al. [24], Manners et al. [26], and Krichen et al. [37].

The difference between diploid and tetraploid subspecies was significant, but greater variation was found among accessions within the same ploidy level. The diploids did not appear grouped separately, unlike the case of the study by Peng et al. [1] of 9 tetraploid and 25 diploid accessions of *Dactylis glomerata* in China, who obtain three separated groups formed two of them by diploids, and the other one by tetraploid accessions. The present results may have been affected by the very small number (2) of *lusitanica* genotypes compared with the other two subspecies studied.

### Cluster analysis

**Fig 1:**
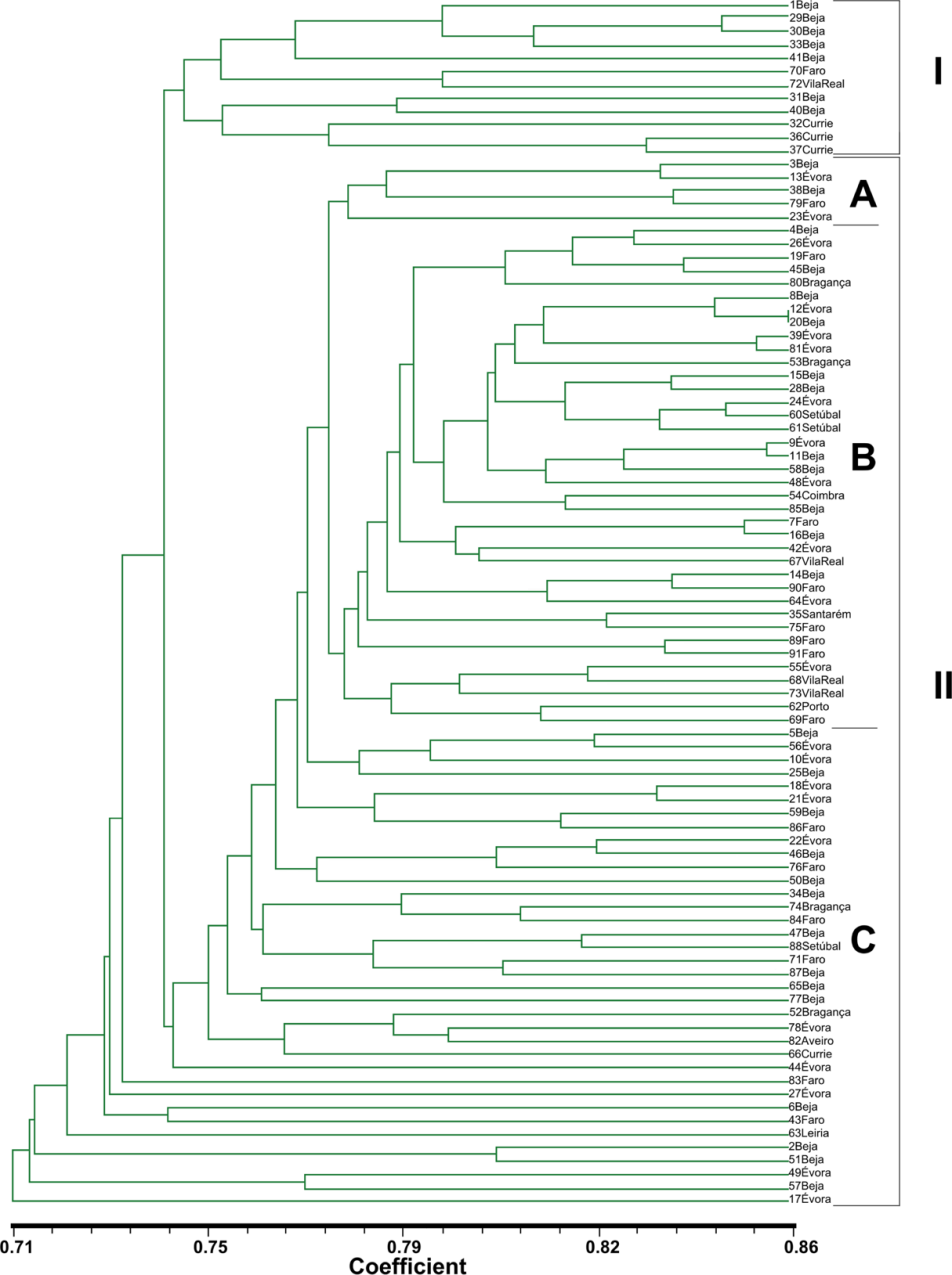
UPGMA dendrogram based on the genetic similarity matrix obtained with the Jaccard index for the data from 288 RAPD+ISSR+AFLP markers.

We first computed the cophenetic correlation coefficients between the similarity matrices and the respective dendrograms (**Table 5**). The values were statistically significant for all markers. Three dendrograms were constructed (**S1–S3 Figs**) to express the results of the cluster analysis of the RAPD, AFLP, and ISSR marker data. Also, from the 288 bands resulting from the analysis of the 3 techniques together, we constructed a dendrogram based on the Jaccard similarity coefficient (**Fig 1**). This dendrogram shows values of the genetic similarity for all the *D*. *glomerata* genotypes varying between 0.71 and 0.86. The genotypes form two main clusters (1 and 2), with the Jaccard index between them being 0.73. Cluster 1 comprises genotypes 1, 29, 30, 33, 41, 70, 72, 31, 40, 32, 36, and 37, all of the same subspecies (*glomerata*). Most of them are from the district of Beja together with those belonging to the Currie commercial variety. Cluster 2 consists of three subgroups (A, B and C). Subgroup 2A is the smallest, comprising genotypes 3, 13, 38, 79, and 23 (three being *glomerata*, and two *hispanica*). Subgroups 2B and 2C are heterogeneous in terms of both geographical origin and subspecies. Subgroup 2B contains 38 accessions, all tetraploid genotypes, and Subgroup 2C contains 36 accessions with 2 diploid genotypes.

**Table 5:** The cophenetic correlation coefficients between the similarity matrices and the respective dendrograms.

Marker	RAPD	AFLP	ISSR	RAPD+AFLP+ISSR
RAPD	**0.680**			
AFLP	0.111	**0.700**		
ISSR	0.165	0.008	**0.780**	
RAPD+AFLP-ISSR				**0.700**

**Fig 2:**
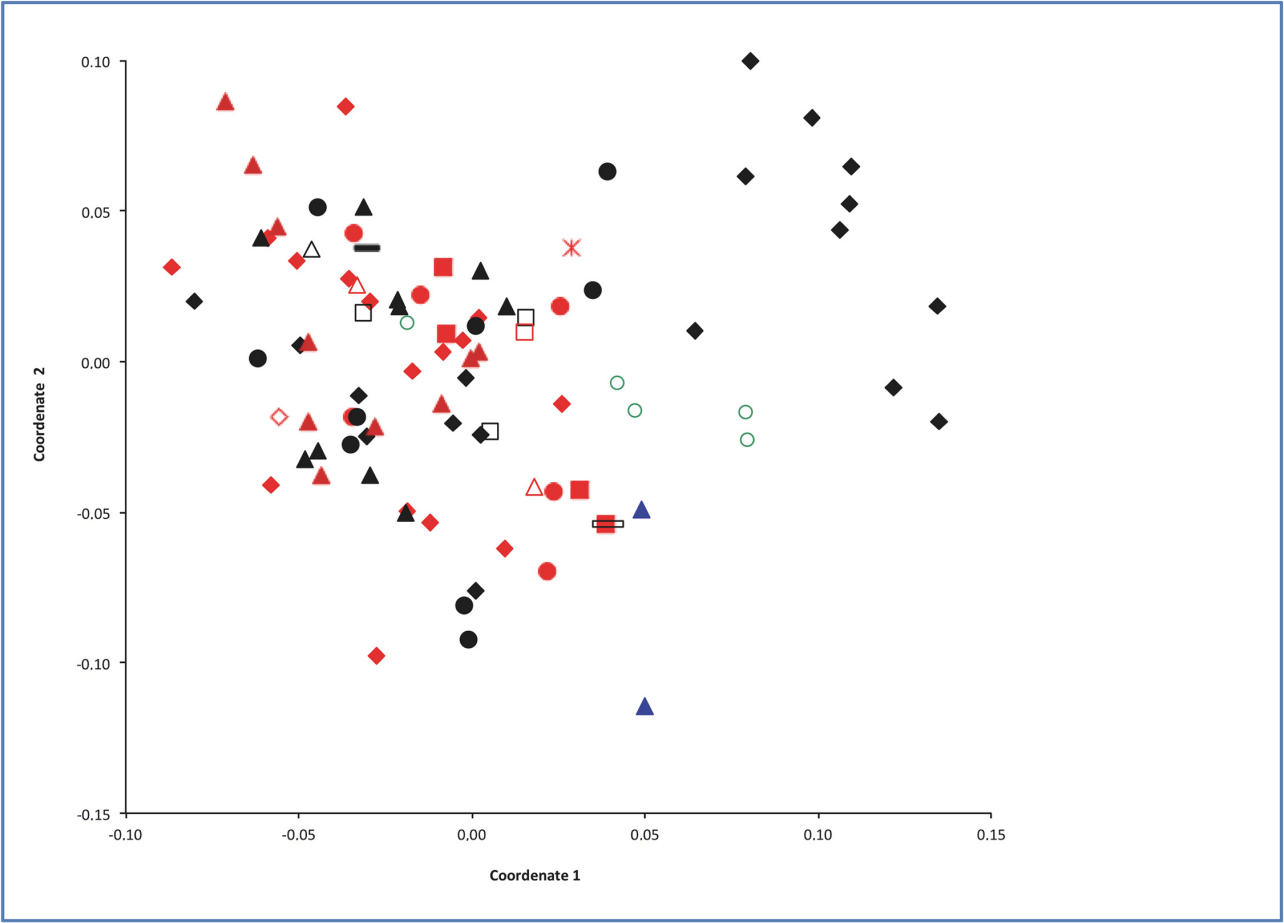
Projection of the genotypes onto the plane defined by the Principal Coordenates 1 and 2, from the data of 288 markers (RAPD, ISSR, and AFLP). Coloration is according to subspecies (black = glomerata, red = hispanica, blue = lusitanica, green = comercial glomerata “Currie”), and symbols indicate the different collection areas (filled: diamonds [Beja], squares [Bragança], triangles [Evora], circles [Faro], line [Leiria], asterik [Santarem]; and, unfilled: squares [VilaReal], circles [Porto], diamonds [Coimbra], triangles [Setubal], line [Aveiro]).

In a principal coordinates analysis performed with the complete set of molecular data (RAPD+ISSR+AFLP) for 91 *D*. *glomerata* genotypes (**Fig 2**), the first two principal coordinates accounted for 8.23% and 6.02% respectively of the total molecular variation. Principal Coordinate 1 separates the genotypes belonging to Group 1 from the other subgroups, with most of its genotypes in the centre of the chart, overlapping significantly with the other genotypes.

Among the main differences found between the techniques studied, there stand out the speed, simplicity, and low cost of RAPD and ISSR in comparison with the more laborious, high cost, use of radioactivity, and time-consuming nature of AFLP [30]. Comparing RAPD with ISSR, one observes that the latter has the capacity to produce more polymorphisms because the primers hardly amplify the non-coding regions of the genome at all, which, as mentioned above, are highly polymorphic. The RAPD technique amplifies both coding and non-coding regions of the genome, but when they amplify in one region they do not amplify in another, thus reducing the possibility of amplifying the most polymorphic regions.

With respect to reproducibility, ISSR was found to be more specific in that it uses larger primers and requires higher alignment temperatures, mitigating the non-reproducibility that is so strongly associated with RAPD [38]. It was also found that the level of polymorphism observed in the collection when the genotypes are studied separately is similar to that observed when the collection is divided into subspecies, as long as the same technique is used. Thus, it is clear that the AFLP technique produces a large number of fragments (bands), as well as a high proportion of polymorphic fragments (90%) compared with the much lower percentages with ISSR (59%) and RAPD (40%). Bahulikar et al. [39] found AFLP analysis to show a greater percentage of polymorphic loci than ISSR analysis. In contrast, Biswas et al. [24] obtained higher levels of polymorphism with SSR than with AFLP, while Krichen et al. [37] found similar levels with the two techniques. The comparison of the capacity to discriminate between genotypes using UPGMA cluster analysis confirmed the findings of Savelkoul et al. [40] in different applications using AFLP. Those authors noted that the technique has good reproducibility and discriminatory power. In the present study, the Jaccard similarity coefficients showed the discriminatory power to decrease in the order AFLP (0.35–0.68) through ISSR (0.74–1.00) to RAPD (0.82–0.99).

In comparing the techniques' correlation coefficients (r), we found the poorest correlation (r = 0.68) to correspond to the dendrogram produced by RAPD markers, it being possible that a distortion had arisen between the original data and the dendrogram. ISSR gave the strongest correlation coefficient (r = 0.78) [38], followed by AFLP (r = 0.70) [37]. Similar values have been reported for *Lolium perenne* using RAPD, ISSR, AFLP, and SSR techniques [41]. Based on the present data, one can conclude that ISSR and AFLP have a greater discriminatory power to reflect genetic relationships among unknown genotypes, and with better reliability than with RAPD. However, considering the three techniques' 288 markers together endowed the dendrogram with added reliability, and the arrangement of the genotypes was similar to that observed with morpho-agronomic data, although the cophenetic correlation continued to be only moderate (r = 0.70). These results are consistent with those reported by Pejic et al. [42] that, in maize, 150 markers are sufficient to reliably estimate genetic similarity. In choosing one of these techniques, it is necessary to weigh their different characteristics with a view to their applicability. Thus, to estimate the genetic diversity in germplasm collections among individuals belonging to the same species or different species, one might choose to apply RAPD [12], [13], [43], but to assess the relationships between individuals that are very close to each other it would be advisable to use the ISSR technique [14], [44]. Finally, using AFLP would allow one to discriminate among, and identify, very close individuals, as when analysing inter- and intra-populational genetic diversity [1], [37] or when the goal is to obtain a greater coverage of the genome [42].

## Conclusions

The molecular markers RAPD, ISSR, and AFLP have been found useful for the study of the genetic diversity of the present *Dactylis glomerata* collection, and the numbers of markers analysed were in all three cases sufficient to discriminate between all genotypes. Of the markers studied, AFLP showed itself to be the most efficient at discriminating genotypes of *Dactylis glomerata* that are genetically closely related, due to its high degree of polymorphism (91%). Although the level of polymorphism revealed by RAPD and ISSR was lower than AFLP (41% and 59%, respectively), these too can be appropriate options since they are easier to implement and less costly.

The study has demonstrated that the 288 markers obtained with the three molecular techniques provide extensive coverage of the genome, and that when we study genotypes close related, the analysis of variability could require more than one DNA-based technique. Finally the data obtained can be used for varietal survey and construction of germplasm collection and providing also additional information that could form the basis for the rational design of breeding programs.

## Supporting Information

S1 Fig UPGMA dendrogram based on the genetic similarity matrix obtained with the Jaccard index for the data from 97 RAPD markers. (TIF)

S2 Fig UPGMA dendrogram based on the genetic similarity matrix obtained with the Jaccard index for the data from 91 ISSR markers. (TIF)

S3 Fig UPGMA dendrogram based on the genetic similarity matrix obtained with the Jaccard index for the data from 100 AFLP markers. (TIF)
