# Supplementary figures and images for: Comparison of RAPD, ISSR, and AFLP Molecular Markers to Reveal and Classify Orchardgrass (Dactylis glomerata L.) Germplasm Variations
Source: PLoS One. 2016 Apr 12;11(4):e0152972. doi: 10.1371/journal.pone.0152972 (PMC4829269; doi:10.1371/journal.pone.0152972)

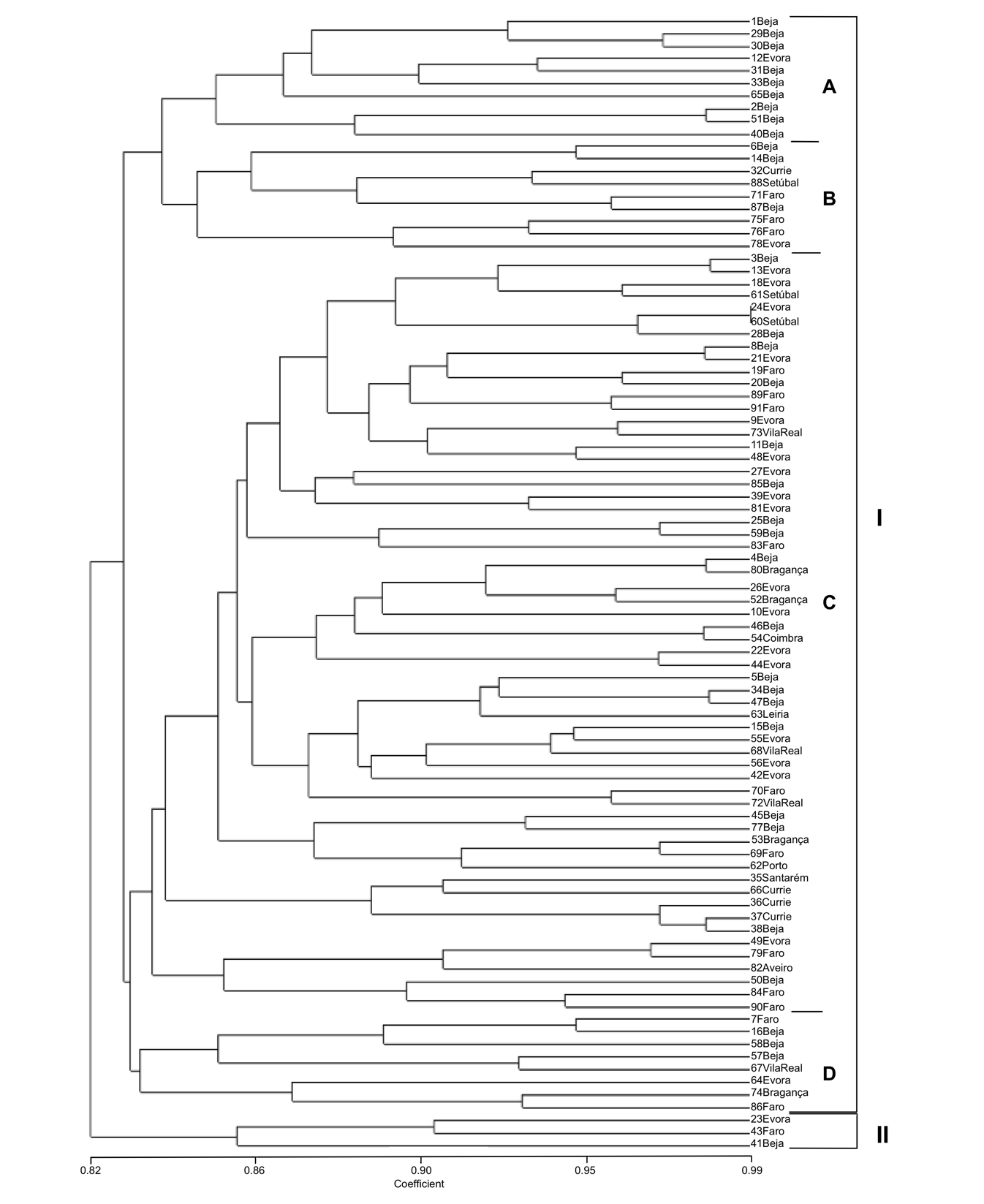

Supplement: S1 Fig — (TIF) [file pone.0152972.s001.tif]

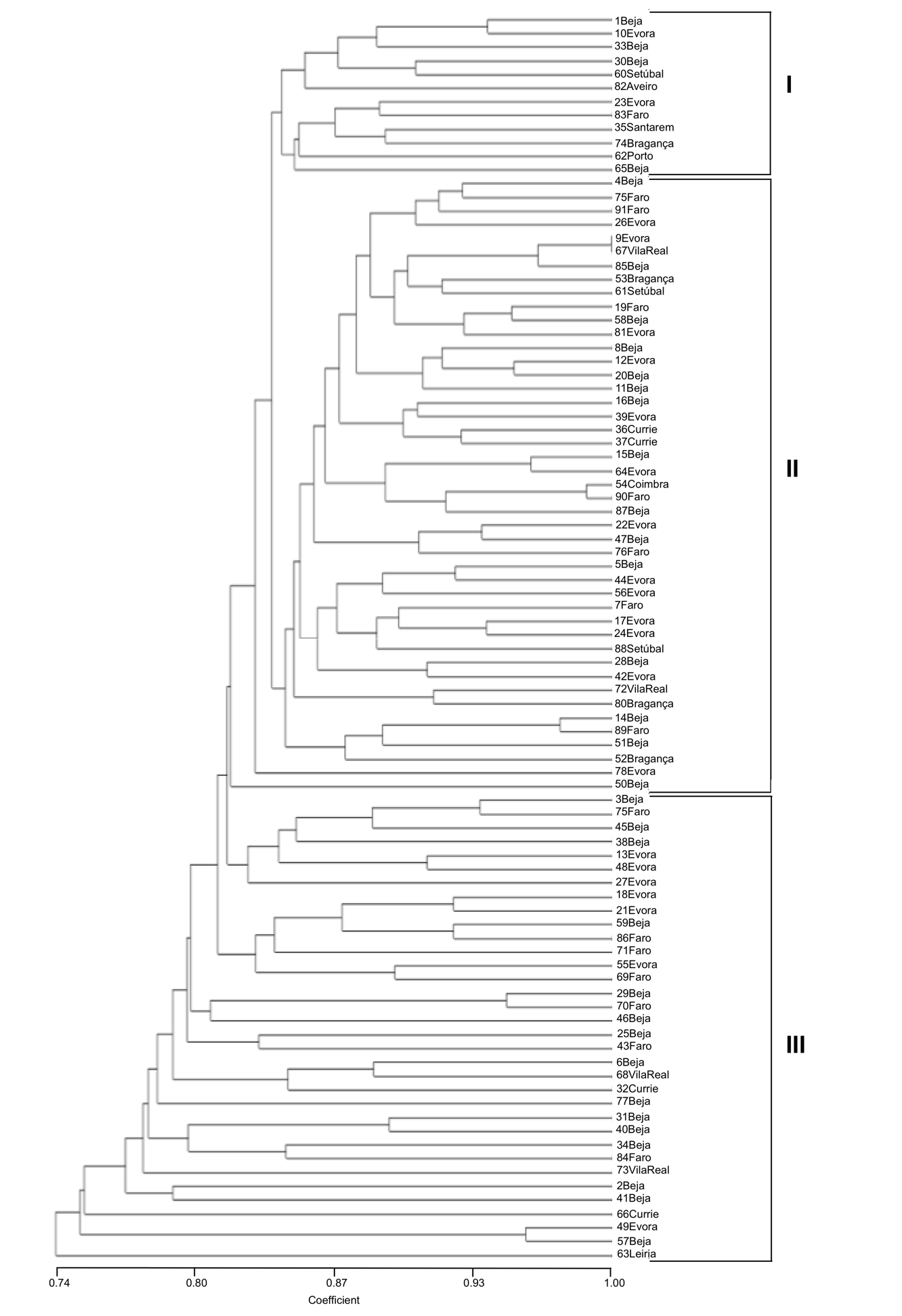

Supplement: S2 Fig — (TIF) [file pone.0152972.s002.tif]

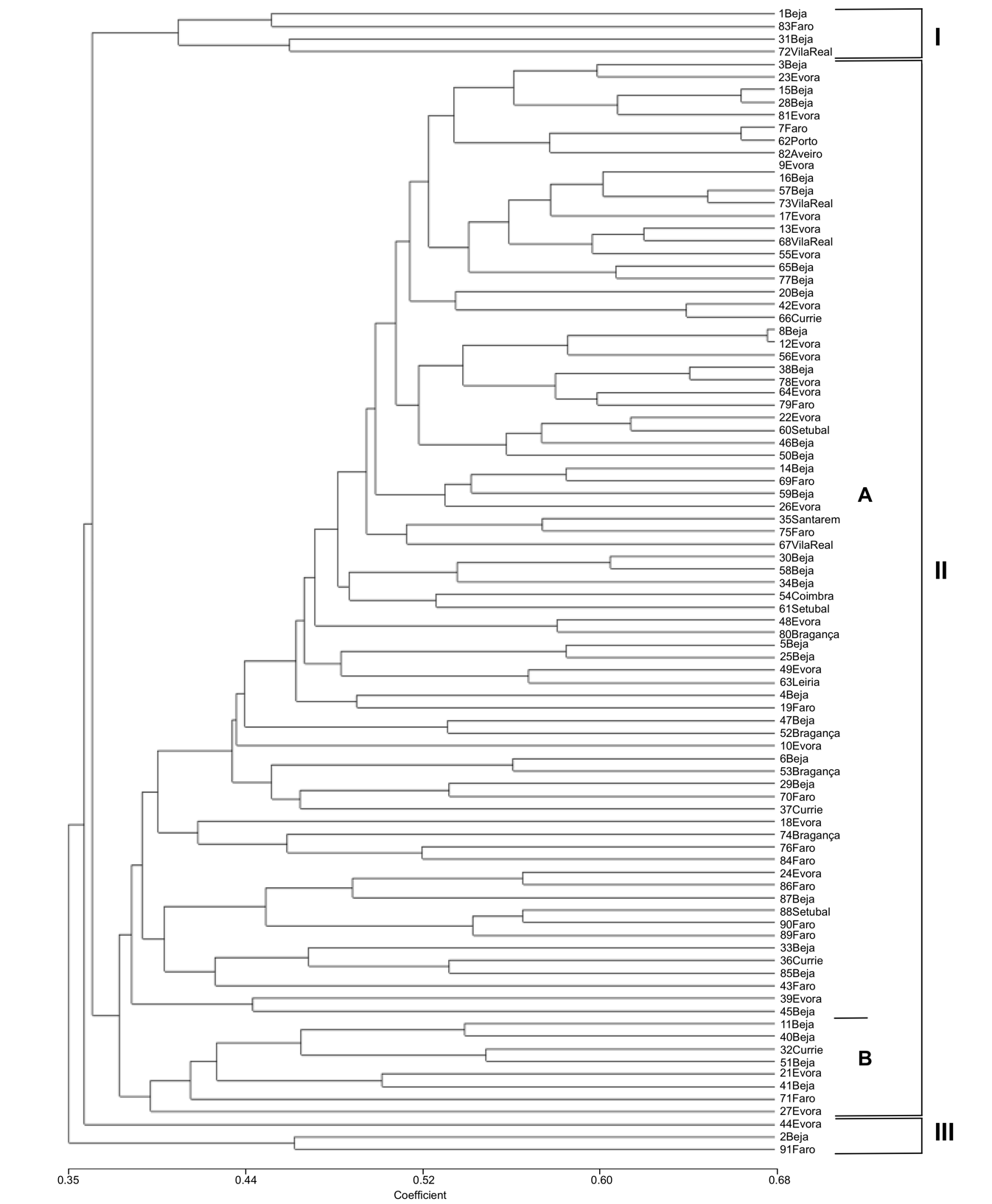

Supplement: S3 Fig — (TIF) [file pone.0152972.s003.tif]
